# Evaluating monitoring methods to guide adaptive management of a threatened amphibian (*Litoria aurea*)

**DOI:** 10.1002/ece3.980

**Published:** 2014-03-19

**Authors:** Deborah S Bower, Evan J Pickett, Michelle P Stockwell, Carla J Pollard, James I Garnham, Madeleine R Sanders, John Clulow, Michael J Mahony

**Affiliations:** School of Environmental and Life Sciences, The University of NewcastleUniversity Dr. Callaghan, Newcastle, New South Wales, 2308, Australia

**Keywords:** Amphibian, auditory, conservation, cost benefit analysis, mark–recapture, tadpole, visual encounter survey

## Abstract

Prompt detection of declines in abundance or distribution of populations is critical when managing threatened species that have high population turnover. Population monitoring programs provide the tools necessary to identify and detect decreases in abundance that will threaten the persistence of key populations and should occur in an adaptive management framework which designs monitoring to maximize detection and minimize effort. We monitored a population of *Litoria aurea* at Sydney Olympic Park over 5 years using mark–recapture, capture encounter, noncapture encounter, auditory, tadpole trapping, and dip-net surveys. The methods differed in the cost, time, and ability to detect changes in the population. Only capture encounter surveys were able to simultaneously detect a decline in the occupancy, relative abundance, and recruitment of frogs during the surveys. The relative abundance of *L. aurea* during encounter surveys correlated with the population size obtained from mark–recapture surveys, and the methods were therefore useful for detecting a change in the population. Tadpole trapping and auditory surveys did not predict overall abundance and were therefore not useful in detecting declines. Monitoring regimes should determine optimal survey times to identify periods where populations have the highest detectability. Once this has been achieved, capture encounter surveys provide a cost-effective method of effectively monitoring trends in occupancy, changes in relative abundance, and detecting recruitment in populations.

## Introduction

Detecting decline in a population is achieved through the identification of a decrease in the population size of a species (Begon et al. 1996). This requires spatial and temporal data to determine the distribution of the population and variability in abundance. When a population size is lowered through a decrease in extent or abundance, it is correspondingly more vulnerable to proximate causes of extinction (Lawton et al. 1994). For threatened populations, prompt identification of population decline is a critical component of successful conservation, and therefore, such populations require ongoing monitoring (Woinarski et al. 2010). Monitoring can also inform managers of the effectiveness of different actions and provides long-term trends to inform research (MacLeod et al. 2011).

Resources are limited, and optimal management should ensure that the costs of monitoring do not override the potential benefits that could be directed into other resources for conservation (Possingham et al. 1993). Different monitoring strategies provide a subset of information, and some methods overlap in the information gained. For example, both capture encounter and auditory surveys are techniques used to count calling frogs, but while the latter method is quicker and easier, it fails to provide the additional information gathered upon sighting and capturing a frog. Therefore, effective management is adaptive and requires consideration of monitoring data to determine the most efficient and effective means of monitoring to ensure that resources are used to their full potential and changes in abundance are detected (Walters and Holling 1990).

Management of habitat and monitoring of the threatened green and golden bell frog (*Litoria aurea*) have been ongoing at Sydney Olympic Park following the discovery of the species during development for the 2000 Olympic games (Darcovich and O'Meara 2008; O'Meara and Darcovich 2008). The conservation of *L. aurea* was legislatively bound to the requirements of the Biodiversity Management Plan, which requires a monitoring program. The annual survival rate of individuals in the population is extremely low (range from 0.06 to 0.44), and consequently, few individuals live past 2 years of age (Pickett et al. 2013). The fast growth rate and early maturity maintain the population with high turnover as most individuals do not live past 2 years (Pickett et al., In press); this increases the importance of consistent breeding events. The small and highly variable population size coupled with low survival means that just two consecutive seasons without breeding could result in a population crash, and therefore, early detection of population decline is crucial.

Long-term monitoring of *L. aurea* at Sydney Olympic Park has provided an extensive data set to compare the value of different monitoring methods. These data can be used to empirically test the effectiveness of monitoring regimes to determine the comparative value and costs. Therefore, we aimed to (1) compare the effectiveness of monitoring methods to detect a decline in the distribution and abundance of *L. aurea* and (2) analyze the cost against the benefit of each method to determine its comparative value as a monitoring tool.

## Methods

### Survey methods

Auditory surveys were completed in 104 ponds within the separate habitat areas known as Narawang Wetland (*n* = 22), Kronos Hill/Wentworth Common (*n* = 37), and the Brickpit (*n* = 45). Surveys were completed three times a year by conducting a single survey at each pond in November, December, and February (replaced by January 2013), from November 2008 to February 2013. An estimate of the number of calling *L. aurea* was recorded upon arrival at each pond for 1 min at which time the call of *L. aurea* was imitated and the number of calling frogs recounted for 1 min.

Capture encounter surveys were completed after each auditory survey and provided a density of frogs per unit of search effort for each pond (number of frogs/search effort); the number of frogs in each pond and total search effort for the precinct were summed to provide a total relative density of frogs for the Brickpit and Kronos Hill/Wentworth Common. These data were also used to provide an estimate of occupancy, defined here as the number of ponds occupied by *L. aurea* during encounter surveys. This was achieved by slowly walking around the perimeter of the pond and searching the emergent vegetation and water, as well as the surrounding terrestrial environment and ground cover. Ponds differed in size so that larger ponds were searched for longer. The number of observers and time spent searching were multiplied, to quantify effort. Upon sighting, each frog was recorded as calling or not, then captured by hand using a thin, disposable plastic bag; the bag was inverted and tied to contain the frog. In addition, distance of the path walked by observers was quantified using Google maps. We also considered noncapture encounter surveys in our cost benefit analysis, although we did not complete surveys without capturing animals, we have included the value of studies where frogs are visually sighted but not captured. This was estimated by removing costs associated with time of capture and processing equipment.

After each survey, *L. aurea* were weighed, measured to the nearest millimeter (snout-to-vent length; SVL) and scanned using a Trovan LID-560ISO pocket reader to detect implanted passive integrated transponder (PIT) tags. In surveys prior to 2010, frogs that were >45 mm snout-vent length and did not have a PIT tag were implanted subcutaneously in the dorsal or dorso-lateral regions (Christy 1996; Gibbons and Andrews 2004). From September 2010, smaller PIT tags were available and frogs between 35 mm and 45 mm were also tagged. Males develop nuptial pads when they reach around 45 mm SVL, so the presence of nuptial pads indicated a male and the absence of this feature indicated a female (Christy 2000). Frogs <45 mm were classed as juveniles. Frogs were released at the site of capture.

Mark–recapture surveys were completed in a spatial cluster of ponds to allow modeling to incorporate potential heterogeneity in recapture rates or individuals and mathematically correct for biases, which might otherwise skew capture or encounter data. Surveys were conducted in a small proportion of the ponds in the Brickpit and Kronos Hill/Wentworth Common that contained the highest relative abundance based on previous surveys from January 2007 (Pickett et al. In press), As such, each cluster of ponds was visited for up to seven consecutive nights, once or twice a season. Population size was estimated using a Lincoln–Petersen index method using one marking and four to six recapture session. Annual mark–recapture surveys occurred in January of each year from 2009 until 2013, and additional surveys were undertaken in September 2010–2012. Occasionally, all ponds within the sampling area were not able to be sampled in one night, and additional nights were required. Consequently, single sample events ranged from one to three nights depending on the number and experience of surveyors.

Mark–recapture surveys were also completed in four ponds within the Kronos Hill precinct in January of each year from 2010 to 2013. Sampling periods lasted one night and were repeated multiple times over consecutive nights. Within-month sampling were conducted at least 1 week after the previous sample for both the Brickpit and Kronos Hill surveys. Processing of frogs was consistent with capture encounter surveys.

Tadpole surveys were completed concurrently with capture encounter surveys and on an extra occasion in January. Minnow traps were tied to emergent vegetation and baited with a yellow glow stick between 15:00 and 20:00 h. The number of traps set in each pond was relative to the size of the pond (between 1 and 50), and additionally, a dip-net was swept through the water for 1 m at a relative number of sweeps at each pond (between 1 and 50). Depth was measured up to 150 cm with premeasured markings on the dip-net in the deepest point of the pond. *Litoria aurea* spend 1.5–11 months as tadpoles (Anstis 2002; Browne et al. 2003); therefore, monthly trapping during the peak of the breeding season was considered adequate to sample breeding events.

### Statistical analyses

We compared the output provided by the different sampling methods in order to test which parameters in the population were correlated and therefore predictive of the other. We include calling male abundance collected during auditory surveys because more males were detected than during encounter surveys. Paired sample *t*-tests were used to compare the number of tadpoles detected in dip nets and minnow traps, the number of frogs detected calling in auditory and encounter surveys, and the number of frogs calling before and after the imitation call. Pearson's correlation tested for correlations between a number of methods, in order to assess whether they were predictive of the other. We tested correlations between: (1) the number of occupied ponds against the relative abundance recorded in capture encounter surveys, (2) the number of occupied ponds, and the number of breeding ponds, (3) the number of males calling in auditory surveys and the relative abundance of frogs, (4) the number of calling males and the number of breeding ponds, and (5) the relative abundance and population size. Frequentist statistics were conducted in spss version 17.0 (IBM SPSS Statistics, Armonk, NY). Population estimates were derived using program mark version 6.1, for detailed methodology see Pickett et al. (In press). Mark–recapture surveys from September and January were compared with encounter surveys in November and February, respectively, except for 2013 when the November encounter survey was used. This exception arose because the visual encounter data detected juveniles from a captive release program and inflated the value considerably; however, these frogs were too small to microchip and were thus excluded from mark–recapture.

To assess the benefit of each additional session and determine the change to the confidence interval provided by additional sessions, models were fitted using all the sessions available (between 5 and 7) and then by removing the last session, rerunning the model and estimating the confidence interval as a proportion of the population size. The cost of each method was estimated by an itemized budget (Data S1); time was estimated from previous sampling occasions.

## Results

### Population performance

**Figure 1:**
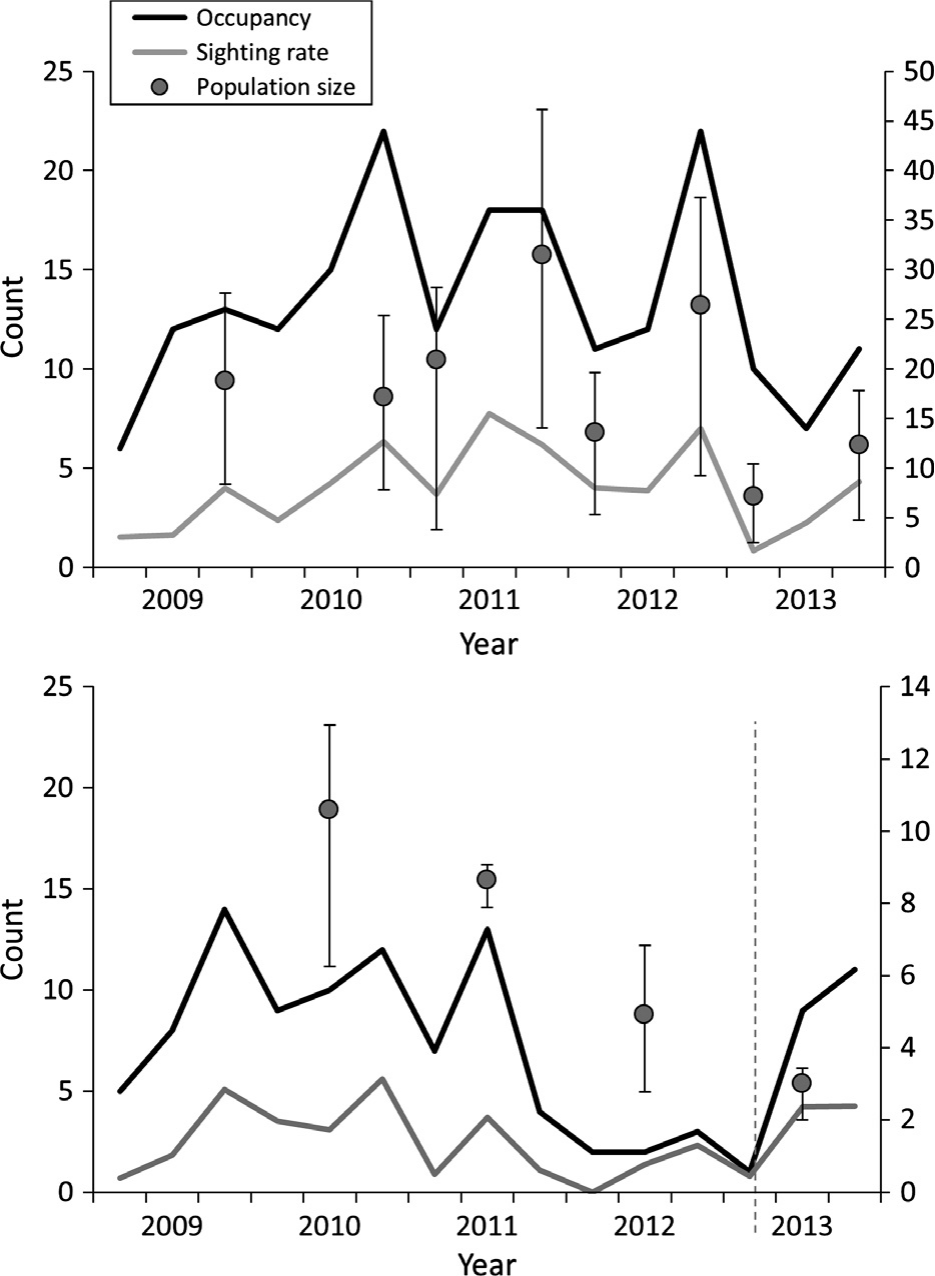
The naïve occupancy and sighting rate (*100) provided by encounter survey data plotted alongside the population size from mark–recapture surveys between 2009 and 2013. All metrics show high variability in the Brickpit over time (top) and a decreasing trend at Kronos Hill/Wentworth Common (bottom) in *Litoria aurea* at Sydney Olympic Park over 5 years. Dashed line indicates a management intervention of releasing 11,500 captive bred tadpoles.

**Figure 2:**
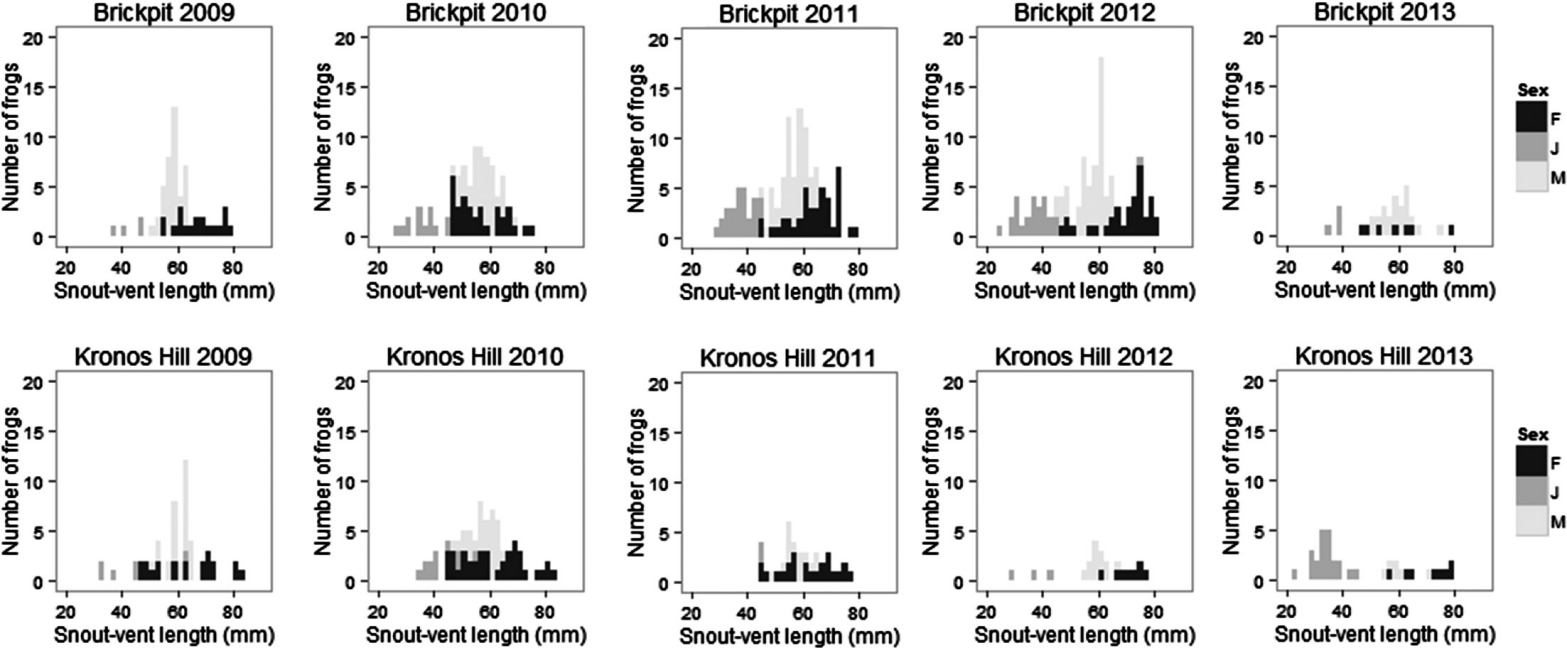
Size class structure of the Brickpit and Kronos Hill/Wentworth Common during capture encounter surveys over 5 years of *Litoria aurea* monitoring at Sydney Olympic Park. The figure depicts the low recruitment in Kronos Hill/Wentworth Common during 2010/2011 and 2011/2012 and the increase in juvenile abundance following a management intervention in the 2012/2013 season. (J, juvenile; M, male; F, female).

The abundance *of L. aurea* varied in the population between 2008 and 2013 (Fig. 1). Population size of *L. aurea* in the Brickpit was variable but did not decrease over time, whereas the population trend in Kronos Hill/Wentworth Common was negative during monitoring. The decline in Kronos Hill/Wentworth Common population size corresponded with a reduction in occupancy from 5 to 15 ponds during the 2008–2010 period, to two to five ponds during surveys in 2011. This decline in occupancy was reflected in the few juveniles seen in the size class structure histogram (Fig. 2) and prompted a captive breed and release management intervention in September 2012 and consequently occupancy increased later in 2013.

### Comparison of methods

**Figure 3:**
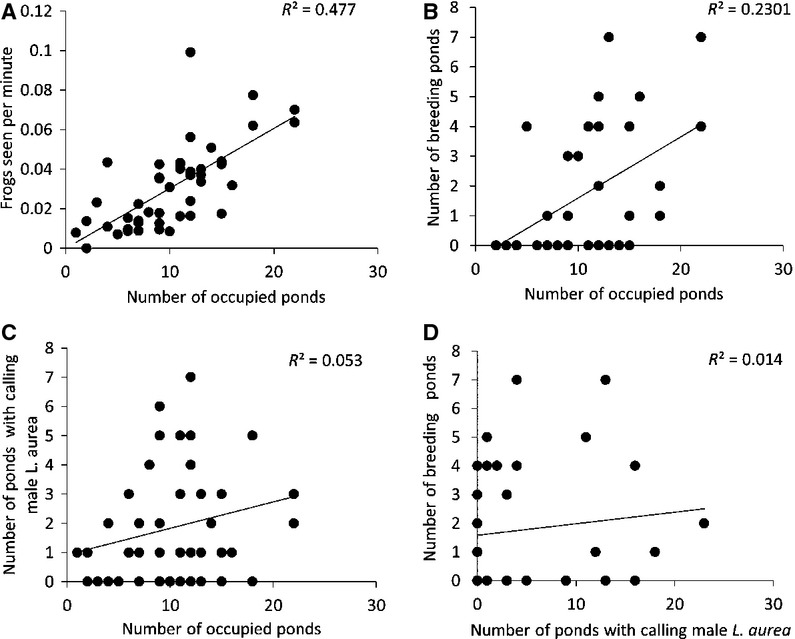
There was a correlation between (A) the relative abundance of frogs in each precinct for each year and the corresponding number of occupied ponds, (B) the number of breeding ponds and the relative abundance of *Litoria aurea*, but not between the (C) number of male *L. aurea* calling during auditory surveys and the occupancy of *L. aurea* recorded in encounter surveys, or (D) the number of calling male *L. aurea* during auditory surveys and the number of breeding ponds detected at Sydney Olympic Park during 2008–2013.

**Figure 4:**
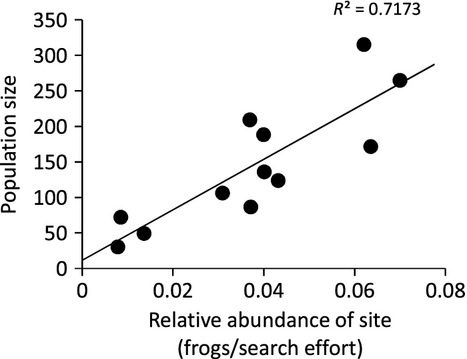
The population size estimated by mark–recapture surveys correlated with the relative abundance of frogs seen in each precinct during encounter surveys between 2008 and 2013 at Sydney Olympic Park.

*Litoria aurea* were distributed throughout more ponds when the relative abundance was higher, and this was clear from the positive correlation between the relative abundance of frogs in each precinct for each year and the corresponding number of occupied ponds (Fig. 3A, Pearson's correlation: *r*^2^ = 0.69, df = 44, *P* < 0.0001). The number of breeding ponds was also correlated with the relative abundance of *L. aurea* (Fig. 3B, Pearson's correlation: *r*^2^ = 0.51, df = 44, *P* < 0.0001). However, male calling obtained during auditory surveys was a poor indicator of relative abundance as it was uncorrelated with the occupancy of *L. aurea* recorded in encounter surveys (Fig. 3C, Pearson's correlation: *r*^2^ = 0.19, df = 44, *P* = 0.19). Calling male abundance also failed to predict breeding distributions as there was no relationship between the number of calling males and the number of breeding ponds (Fig. 3D, Pearson's correlation: *r*^2^ = 0.09, df = 44, *P* = 0.54; Fig. 3D). Relative abundance indicated population size; there was a positive correlation between the number of frogs seen per minute during capture encounter surveys and population size from mark–recapture surveys (Pearson's correlation: *r*^2^ = 0.72, df = 11, *P* < 0.05, Fig. 4).

Trapping detected tadpoles in 64 ponds between 2008 and 2012, whereas dip-net surveys only detected tadpoles in 16 ponds. In the ponds, where tadpoles were detected, a significantly higher number of tadpoles were captured per trap, than per dip-net sweep (Paired sample *t*-test, *t* = 2.96, df = 21, *P *<* *0.05). There were no sessions when dip-net surveys detected more *L. aurea* tadpoles than traps.

Auditory surveys detected calling *L. aurea* in 83 ponds between 2008 and 2013. This was significantly higher than the 56 ponds that detected calling frogs during encounter surveys (Paired sample *t*-test, *t* = 2.86, df = 44, *P *<* *0.05). The mean number of *L. aurea* calling after the imitation call (1.92 ± 0.20) was significantly higher than before (1.35 ± 0.19; paired sample *t*-test, *t* = 2.84, df = 100, *P *<* *0.005).

### Comparison of surveys occasions

A single encounter survey containing one survey at each pond provided an average of 58% of the occupancy information that was collecting during three repeated surveys (Table 1). Surveys conducted during February on average provided the highest estimate of occupancy out of the three monthly surveys. The information gathered during two cumulative encounter surveys increased estimates to 84% of the occupancy information collected during three repeat surveys. The 2-month cumulative occupancy estimate was highest using February and December surveys (91%) for all years except 2012 (Table 1).

**Table 1:** The proportional contribution of (A) encounter surveys to total occupancy of ponds (B) ponds occupied by tadpoles and calling males, during 2009–2013 at Sydney Olympic Park

Session	2009 (%)	2010 (%)	2011 (%)	2012 (%)	2013 (%)
*(A)*					
November	35	57	44	38	40
December	69	57	73	51	52^1^
February	75	83	54	76	69
November + February	88	93	75	93	86
December^1^ + November	71	75	87	76	71^1^
February + December^1^	98	95	94	84	86^1^
*(B)*					
Proportion of ponds occupied by tadpoles					
November	4	59	25	50	50
December	6	45	38	63	33^1^
February	0	59	38	88	10
Proportion of ponds with calling males during auditory survey					
November	26	64	87	38	22
December	63	27	40	77	44^1^
February	11	36	0	23	78

1 Note that in 2013, the December session is replaced by a January session.

**Figure 5:**
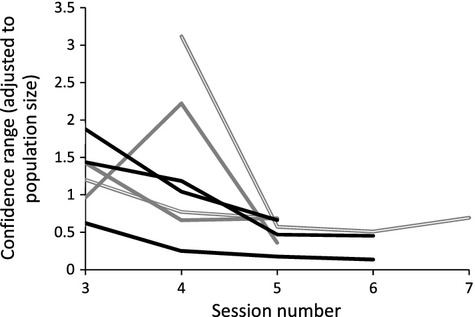
The confidence range decreases as the number of recapture sessions increase using mark–recapture data in *Litoria aurea* at Sydney Olympic Park. (Brickpit shown in gray, Kronos Hill/Wentworth Common in black, January sessions are bold and September sessions are empty lines).

The number of calling males detected during auditory surveys within any given month was highly variable and estimated between 0% and 87% of the total number of ponds observed with calling during the season. The number of calling males was not consistently higher or lower in any particular month. Similarly, the number of ponds where breeding was detected from tadpole surveys was relatively similar among all months. Confidence intervals from mark–recapture sessions (adjusted to population size) usually converged in the fourth or fifth session (Fig. 5).

Capture encounter surveys required the most funding and ranked second in time resources; they allowed an estimation relative abundance, distribution, and recruitment of frogs throughout Sydney Olympic Park (Table 2). Mark–recapture surveys were similar to capture encounter surveys in cost and effort but required specialized analytical skills; they provided a robust population estimate of a subset of ponds and have the capacity to estimate survival and immigration. Noncapture encounter surveys were less costly in time in effort but did not provide information on recruitment rates. Tadpole surveys took the most time to complete and were high in cost, although they did not require a high level of analytical skill. Tadpole surveys provided an indication of the complete distribution of breeding ponds but only correlate weakly with occupancy of frogs. Dip-net surveys and auditory surveys were quick and cheap by comparison and required moderate and little skill, respectively, but did not provide information that accurately reflected the abundance or occupancy estimated by mark–recapture or encounter surveys.

**Table 2:** Financial cost and people hours for one complete *Litoria aurea* survey at Sydney Olympic Park

Survey type	Initial cost (AU $)^1^	People hours	Level of difficulty
Noncapture encounter	1214.7	72	1
Capture encounter	2551.7	144	2
Mark–recapture	2551.38	144	3
Auditory	814	20	1
Tadpole trapping	2137.45	42	2
Tadpole dip-net	129.40	21	2

Requires very little prior knowledge or training. For example, Can be collected by Community Group. Requires skill or training in data collection. For example, Can be completed by personnel trained in collection of scientific data collection such as a graduate student. Requires skill in experimental design, data collection, and complex analysis. For example, Requires data analysis at postgraduate level.

1 See Data S1 for calculation of cost and time estimate.

## Discussion

The primary goal of monitoring is to improve current monitoring strategies and to inform management decisions (McDonald-Madden et al. 2010). Encounter surveys were the only methods to provide an indication of the overall distribution of *L. aurea*. The relative abundance data strongly correlated with the population size estimates obtained through mark–recapture analyses suggesting that encounter survey is a suitable method to broadly indicate changes in abundance. While mark–recapture analyses are crucial to understand population functioning by providing information such as survival, immigration, and recapture rate, they are rigid in their requirements to validate assumptions and must meet a minimum proportion of recapture for models to converge (Pollock and Otto 1983). Conversely, encounter surveys were informative in low densities and even in the absence of frogs. However, noncapture encounter surveys failed to identify recruitment into the population, which is a crucial annual performance indicator of future changes to distribution and abundance in the population.

Encounter surveys were less efficient at identifying calling males than auditory surveys. However, this metric was uninformative to managers because the number of ponds with calling males detected in the auditory survey failed to predict the level of breeding and did not correlate with overall occupancy of *L. aurea* measured in encounter surveys. Male calling is used to estimate abundance in other species (Driscoll 1998) but is not valid as a metric of population performance or breeding levels in *L. aurea* with the survey method we used. Some species of frogs are more detectable using automated sound devices (Bridges and Dorcas 2000; Hsu et al. 2005; Acevedo and Villanueva-Rivera 2006), but this method is unlikely to be feasible for monitoring many sights in close proximity. Alternatively, tadpole trapping was a successful means of identifying breeding in ponds and correlated with the overall occupancy of ponds. However, tadpole trapping ranked high in cost and time (Table 3), and its goal – to determine recruitment – could be easily replaced by analyzing the size class structure of frogs sampled during capture encounter surveys. It is also important to note that death in the tadpole phase is high (Licht 1974) and knowledge of the abundance of juveniles is more informative to assess recruitment to the population.

**Table 3:** Comparative value and informative capacity of different monitoring strategies used for *Litoria aurea* at Sydney Olympic Park 2008–2013

Monitoring method	Time required	Expertise	Cost	Population abundance	Occupancy/Distribution	Survival	Population structure	Movement	Recruitment	Calling distribution
Noncapture encounter survey	Low	Low	Low	No	Yes	No	No	No	No	Yes
Capture encounter survey	Medium	Medium	Medium	Yes	Yes	No	Yes	Yes	Yes	Yes
Mark–recapture	Very high	High	High	Yes	No	Yes	Yes	Yes	Yes	No
Auditory	Low	Low	Low	No	No	No	No	No	No	Yes
Tadpole surveys	High	High	High	No	No	No	No	No	Yes	No

Encounter surveys were most effective at quantifying occupancy in February and December. Combining these two surveys provided occupancy estimates of 84–90% of the total estimated occupancy using three sessions. This suggests that a third survey in November contributes comparatively little information to knowledge of occupancy and distribution, although it may be important for other purposes (such as identification of early season breeding and density of frogs following winter, when chytrid load is likely to be highest). Encounter surveys have the additional benefit of sampling all the species in a site, assessing multiple population trends simultaneously and opening the possibility for analysis of community interactions (Hutchens and DePerno 2009). However, encounter surveys are not a reliable function of population size for all species of amphibians (Funk et al. 2003) and can be fraught with bias that mislead population trends, particularly when detection levels are heterogeneous (Mazerolle et al. 2007), therefore the relationship between encounter survey estimates and population size should be thoroughly assessed prior to use. Alternatively, tadpole surveys and calling surveys were not consistently high or low in any given month suggesting that reproduction is driven by an unexplained factor such as behavior or climatic variation. Mark–recapture models converged with tight confidence intervals after four or five sessions. To minimize cost and effort, surveyors should model the data after each session to determine whether further sessions are necessary to tighten confidence intervals.

Conservation regimes should aim to ensure the persistence of the population through (1) adaptive management and associated monitoring and (2) triggering an action plan if crucial population parameters fail. Parameters that trigger action should include a lack of recruitment or a decline in occupancy or abundance beyond the norm. The only method to identify all three criteria in our study was capture encounter surveys. However, capture encounter surveys alone are unlikely to provide managers with the knowledge to implement the optimal management action. Saving valuable resources by streamlining monitoring methods and redirecting resources toward adaptive management is likely to provide far more conservation benefit to the species (McDonald-Madden et al. 2010). Future monitoring of populations that display characteristics with low abundance and minimal resources should aim to determine optimal survey time to capture periods of the highest detectability, occupancy, and recruitment into the population. Once this has been achieved, capture encounter surveys can provide a cost-effective method of monitoring populations.

## Supporting Information

Data S1 Budget: itemized budget of core requirements for each monitoring method used for *L. aurea* surveys at Sydney Olympic Park. Each method assumes a team of four people completing one survey period based from the Sydney Olympic Park lodge.
