# Supplementary material for: Evaluating monitoring methods to guide adaptive management of a threatened amphibian (Litoria aurea)
Source: Ecol Evol. 2014 Mar 19;4(8):1361–8. doi: 10.1002/ece3.980 (PMC4020695; doi:10.1002/ece3.980)
Supplement: Data S1 — Budget: itemized budget of core requirements for each monitoring method used for L. aurea surveys at Sydney Olympic Park. Each method assumes a team of four people completing one survey period based from the Sydney Olympic Park lodge. [file ece30004-1361-sd1.docx]

Supplementary material

Budget: Itemised budget of core requirements for each monitoring method used for *L. aurea* surveys at Sydney Olympic Park. Each method assumes a team of four people completing one survey period based from the Sydney Olympic Park lodge.

|  | Time | Initial cost | Ongoing cost (following season) |
| --- | --- | --- | --- |
| Non-capture encounter survey | 6 hr*2 ppl*6 nights = 72 hours | Waders ($100*4)=$400  Torch/batteries($200*4)=$800  Vehicle: $70c/km * 21km = $14.70 | Batteries $24.49(40pk)  Replacing waders $400 |
| Capture encounter survey | 6 hr*4 ppl*6 nights =144 hours | Waders ($100*4)=$400  Torch/batteries($200*4)=$800  Microchips ($3.64*250)=$912  Trovan=$425  Plastic bags ($2.35*4)=9.36  Vehicle: $70c/km * 21km = $14.70 | Batteries $24.49(40pk)  Replacing waders $400  Plastic bags ($2.35*4)=9.36 |
| Mark-recapture survey | 6 hr * 4 ppl * 1 night  = 24 hours/day (144 max) | Waders ($100*4)=$400  Torch/batteries($200*4)=$800  Trovan=$425  Microchips ($3.64*250)=$912  Plastic bags ($2.35*4)=9.36  Vehicle: ($70c/km*51km)=$35.70max | Microchips (250) $912  Plastic bags ($2.35*4)=9.36  Batteries $24.49(40pk) |
| Auditory | 8 min*150 ponds = 20 hours | Torch/batteries($200*4)=800  Vehicle: ($70c/km*21km)=$14.70 | Batteries $24.49(40pk) |
| Tadpole trapping | 6 hr*7 days  = 168 hours | Minnow traps ($8*100)=$800.00  Holding tubs ($17.41*5)=$87.05  Glow sticks (67c*1800)=$1221.00  Vehicle: ($70c/km*21km)=$29.40 | Glow sticks*1800=$1221 per season |
| Tadpole Dip-net surveys | 3 hr*7 days  = 21 hours | Dip-net ($50*2)=$100.00  Vehicle:($70c/km*21km)=$29.40 | 0 |

*Optional costs*

*Costs distributed to all tasks:* Personal protective equipment (raincoat, suncream, first aid kit),Waterproof paper, Pencil, Clipboard, GPS, Flagging tape, Water quality meter, Kestral, Callipers, Pesola scales, Chytrid swabs, Bleach for cleaning waders and traps. Optional research equipment not included: Ethanol, Chytrid swabs, Pesola scales, Callipers, Depth stick, water quality meter, ibuttons, genetic analysis.
